# ELIXIR biovalidator for semantic validation of life science metadata

**DOI:** 10.1093/bioinformatics/btac195

**Published:** 2022-04-05

**Authors:** Isuru Liyanage, Tony Burdett, Bert Droesbeke, Karoly Erdos, Rolando Fernandez, Alasdair Gray, Muhammad Haseeb, Simon Jupp, Flavia Penim, Cyril Pommier, Philippe Rocca-Serra, Mélanie Courtot, Frederik Coppens

**Affiliations:** European Molecular Biology Laboratory, European Bioinformatics Institute (EMBL-EBI), Hinxton CB10 1SD, UK; European Molecular Biology Laboratory, European Bioinformatics Institute (EMBL-EBI), Hinxton CB10 1SD, UK; Department of Plant Biotechnology and Bioinformatics, Ghent University, 9052 Ghent, Belgium; VIB Center for Plant Systems Biology, 9052 Ghent, Belgium; European Molecular Biology Laboratory, European Bioinformatics Institute (EMBL-EBI), Hinxton CB10 1SD, UK; European Molecular Biology Laboratory, European Bioinformatics Institute (EMBL-EBI), Hinxton CB10 1SD, UK; Department of Computer Science, Heriot-Watt University, Edinburgh EH14 4AS, UK; European Molecular Biology Laboratory, European Bioinformatics Institute (EMBL-EBI), Hinxton CB10 1SD, UK; European Molecular Biology Laboratory, European Bioinformatics Institute (EMBL-EBI), Hinxton CB10 1SD, UK; European Molecular Biology Laboratory, European Bioinformatics Institute (EMBL-EBI), Hinxton CB10 1SD, UK; INRAE, BioinfOmics, Plant Bioinformatics Facility, Université Paris-Saclay, 78026 Versailles, France; INRAE, URGI, Université Paris-Saclay, 78026 Versailles, France; Department of Engineering Science, University of Oxford e-Research Centre, University of Oxford, Oxford OX1 3QG, UK; European Molecular Biology Laboratory, European Bioinformatics Institute (EMBL-EBI), Hinxton CB10 1SD, UK; Ontario Institute for Cancer Research, Toronto, ON M5G 0A3, Canada; Department of Plant Biotechnology and Bioinformatics, Ghent University, 9052 Ghent, Belgium; VIB Center for Plant Systems Biology, 9052 Ghent, Belgium

## Abstract

**Summary:**

To advance biomedical research, increasingly large amounts of complex data need to be discovered and integrated. This requires syntactic and semantic validation to ensure shared understanding of relevant entities. This article describes the ELIXIR biovalidator, which extends the syntactic validation of the widely used AJV library with ontology-based validation of JSON documents.

**Availability and implementation:**

Source code: https://github.com/elixir-europe/biovalidator, Release: v1.9.1, License: Apache License 2.0, Deployed at: https://www.ebi.ac.uk/biosamples/schema/validator/validate.

**Supplementary information:**

Supplementary data are available at *Bioinformatics* online.

## 1 Introduction

Today’s genomics data ecosystem has been described as a ‘Tower of Babel’, due to an ever-increasing amount of data generated, using different technologies, in a widening number of domains, hosted in a constantly growing number of databases. This massive diversification makes data science an extremely labour intensive and thus a costly undertaking. Data FAIRification (Wilkinson *et al.*, 2016) aims at addressing those challenges by promoting adherence to a set of principles that facilitate data reuse and interoperability. Validation of metadata describing biomedical entities is a crucial part of this process. However, rules for validation are often hard coded in specific resources, and not shared efficiently. Moreover, checklists such as those used by archives (Harrison *et al.*, 2021) can still lead to various interpretations and diverging implementations, resulting in data heterogeneity which prevents its efficient reuse. Therefore, next to clear documentation of best practices, real-world implementations of tools enforcing shared validation processes are needed.

JavaScript Object Notation (JSON) is an IETF standard specifying a lightweight data interchange format. JSON Schema is a vocabulary to specify the structure of a JSON document. Both JSON and JSON Schema are extensively used for data exchange, APIs and standard definitions. Whilst JSON Schema provides a comprehensive vocabulary to validate the structure and the syntax of a JSON document, it contributes little to checking semantics of the content. In life sciences, compliance to metadata schemas often mandates assessing if a value adheres to specified ontologies—e.g. check that the value of a ‘disease’ attribute is a subclass of a disease ontology term. To ensure high-quality metadata, such strict validation checks are required, specifically via queries based on the ontology structure itself. To address this, we have extended the JSON Schema vocabulary with custom keywords that describe how a particular property constrained to an ontology term identifier should be validated. This paper describes how we deployed the ELIXIR biovalidator and applied it to plant related use cases to enhance FAIRness of the data collected and submitted to public archives.

## 2 Implementation

We have developed the ELIXIR biovalidator, a tool for validating life sciences metadata, encoded as JSON documents, against declarative metadata standards that are encoded as JSON Schema. The ELIXIR biovalidator is based on the widely used Ajv JSON Schema validator (Poberezkin, 2021). Through the addition of validation rules for user-defined keywords, we have augmented the validator with ontology-based constraints, such as *isValidTerm* to check if a given ontology term exists in the EMBL-EBI Ontology Lookup Service (OLS) (Jupp *et al.*, 2015). At the time of writing, the ELIXIR biovalidator supports four extended keywords for ontology and taxonomy validation (elixir-europe, 2021). These four keywords enable different modalities of ontology-based validation against any class in the OLS. For example, the keyword *graph_restriction*, used with a parent term ID and an ontology ID, allows us to express that a JSON property such as *disease_ontology_id* can only have terms that are from the Phenotype and Trait Ontology (PATO) or Monarch Disease Ontology (MONDO). Furthermore, these terms must be a subclass of the disease classes *PATO:0000461* or *MONDO:0000001*.

The ELIXIR biovalidator is capable of running as a service or as a one-time script to validate a given JSON document against a schema (elixir-europe, 2021). When run as a service, users can validate using the web interface or an API, which is more suited for batch validations. A Docker image is available for testing in a local environment. The biovalidator is currently deployed in the data ingest system for the Human Cell Atlas project as well as the EMBL-EBI BioSamples (Courtot *et al.*, 2022), where it was used to ensure compliance of over 18 million samples to multiple checklists, such as MiXS and MIAPPE [Minimal Information about a Plant Phenotyping Experiment (Papoutsoglou *et al*., 2020)] for genomic and plant metadata, respectively.

## 3 Validation of plant metadata

**Fig. 1.**
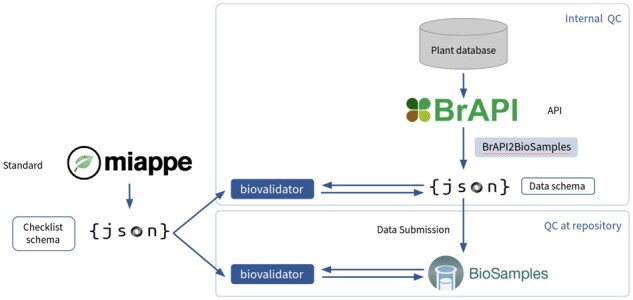
Data validation for the plant use case. A data submitter uses an institutional data repository as a broker to submit Biosamples metadata through the API, which is validated against the MIAPPE JSON Schema. This metadata from the plant phenotyping databases is exposed through the Breeding API (BrAPI) and formatted using the BrAPI2Biosamples script to JSON objects. These objects can be validated using the ELIXIR biovalidator against an MIAPPE JSON Schema checklist

Plant research institutes across the globe have developed databases and tools to manage and store plant phenotyping data, tailored to their specific use cases. MIAPPE is an open, community driven metadata standard that adequately describes plant phenotyping experiments. The Breeding API [BrAPI (Selby *et al.*, 2019)] was developed synergistically with MIAPPE to provide a common, programmatic interface ensuring databases and tools interoperability through the use of a common metadata representation; BrAPI is therefore a web service API implementation of MIAPPE. This standardized API enables the development of scripts that work on all BrAPI-enabled plant phenotyping databases. One such script, BrAPI2Biosamples, can be used to export JSON objects using the MIAPPE nomenclature (Supplementary material). The ELIXIR biovalidator can validate these objects of (user-provided) metadata for high-quality FAIR data for plant phenotyping. The ontology validation ensures semantic validity of any ontology terms present in MIAPPE-compliant data. This also facilitates the submission of MIAPPE-compliant data to BioSamples, as the same validator is used by BioSamples for validating sample metadata either before or at the submission time (Fig. 1). The development of an independent module allowed for the integration of the ELIXIR biovalidator into the BrAPI ecosystem. In the future, we will also implement the validation in data management platforms such as FAIRDOM/SEEK (Wolstencroft *et al.*, 2015) and the ISA (Johnson *et al.*, 2021) model and its JSON Schema definition.

## 4 Conclusion

The ELIXIR biovalidator allows to verify compliance of both the structure and content of JSON documents by extending the existing JSON Schema syntax. The biovalidator is capable of validating ontology terms embedded in JSON documents against requirements. Enabling this quality control for community standards is crucial to develop semantic interoperability in a distributed ecosystem of FAIR digital objects, as envisioned in the European Open Science Cloud Interoperability Framework (Corcho *et al.*, 2021). In the future, we plan to further extend the biovalidator by adding support for identifier cross-reference checking by integrating it with Identifiers.org (Juty *et al.*, 2012). This will enable the biovalidator to check the validity of accessions present in the JSON data.

## Supplementary Material

btac195_Supplementary_Data
