# Correction to: ELIXIR biovalidator for semantic validation of life science metadata

**DOI:** 10.1093/bioinformatics/btac835

**Published:** 2023-01-05

This is a correction to: Isuru Liyanage, Tony Burdett, Bert Droesbeke, Karoly Erdos, Rolando Fernandez, Alasdair Gray, Muhammad Haseeb, Simon Jupp, Flavia Penim, Cyril Pommier, Philippe Rocca-Serra, Mélanie Courtot, Frederik Coppens, ELIXIR biovalidator for semantic validation of life science metadata, *Bioinformatics*, Volume 38, Issue 11, 1 June 2022, Pages 3141–3142, https://doi.org/10.1093/bioinformatics/btac195

In the originally published version of this manuscript, the acknowledgement section was incomplete. The authors acknowledge Danielle Welter’s contribution to the conception and development of the graph restriction framework.

This error has been corrected online.

