# Supplementary material for: ELIXIR biovalidator for semantic validation of life science metadata
Source: Bioinformatics. 2022 Apr 5;38(11):3141–2. doi: 10.1093/bioinformatics/btac195 (PMC9154242; doi:10.1093/bioinformatics/btac195)
Supplement: btac195_Supplementary_Data [file btac195_supplementary_data.pdf]

Supplementary Material

BrAPI2BioSamples
Manual ENA submissions are only possible through the API with existing, presubmitted BioSamples IDs. The BrAPI2BioSamples Python command line tool does that presubmission by (i) extracting Biosample metadata from a BrAPI endpoint before (ii) submitting the metadata using the Biosamples API and (iii) giving back the list of Biosamples ID with corresponding BrAPI ID. The tool can be installed using the python package manager pip:

```
pip install brapi2biosamples
```

The BrAPI endpoint is required to have the following calls implemented:

```
- /trials/{trialDbId}
- /studies/{studyDbId}/germplasm
- /germplasm/{germplasmDbId}
```

The BrAPI2BioSamples tool allows submissions of samples from phenotyping platforms containing the metadata to BioSamples, ensuring samples are MIAPPE-compliant. The tool can also be used to update existing samples in BioSamples to provide additional metadata. When metadata is not available on the BrAPI endpoint, it can be injected using a supplementary metadata yaml file. Upon first use it is recommended to run the script in draft mode to check that all the data is parsed correctly; later on a submission can be done on the dev instance or the production instance of Biosamples.

Here is the minimal command to use the tool:

```
brapi2biosamples -t 1 -e "https://pippa.psb.ugent.be/BrAPIPPA/brapi/v1" -D
--draft "self.pippa_submission"
```

The explanation of these parameters and options are given below:

Option	Description	Required
-v, --version	Print version number	
-t, --trialDbId	The identifier of a trial	yes
-e, --endpoint	The URL towards the BrAPI endpoint, not ending with /	yes
-d, --date	The date of sample publication (example:2021-01-20T17:05:13Z)	
-D, --domain	The domain of your ENA account	yes
--draft	When this flag is given, the samples will not be submitted to BioSamples but exported as JSON	
--dev	When this flag is given, the samples will be submitted	

	to the dev instance of BioSamples	
--secret	Path to a secret.yml file to deliver the BioSample credentials	
--update	Path to a tsv file to update submissions	
--info	Path to a info.yml file to deliver the extra attributes	
--output	Path to a directory where the JSON files are written to.	
-h, --help	Show this message and exit.	
--rename	If the "germplasmDbId" is source specific, reconstruct the name with "genus, instituteName and accessionNumber".	
--decode	Specify the fields that need to be decoded by base64, split by comma. ex -c "field 1, field 2"	

The output is a tab separated file with two columns: germplasmDbId and the Biosamples accession number. This allows traceback of the submitted samples. This tab separated file can also be used to update the samples later on if needed.

This tool was used to successfully submit and validate around 500 maize samples from the PIPPA phenotyping platform to BioSamples.

Testing ELIXIR biovalidator deployed at EBI BioSamples

The ELIXIR biovalidator is deployed at EBI BioSamples for sample validation. The `` `/validate` `` endpoint can be used to validate given JSON data against JSON Schema.

Following is an example request to validate whether the `disease_id` field in a given JSON document is a subclass of `MONDO:0000001` or `PATO:0000461`. In the example we pass a invalid ontology term for the `disease_id` field and get a validation error message.

```
Request URL: http://localhost:3020/validate
Request Method: POST
Content-Type: application/json; charset=utf-8
Payload:
{
  "schema": {
    "$schema": "http://json-schema.org/draft-07/schema#",
    "type": "object",
    "properties": {
      "disease": {
        "description": "The disease for the sample species.",
        "type": "string"
      },
      "disease_id": {
        "description": "The ontology id for the disease sample species.",
        "type": "string",
```

```
        "graph_restriction": {
          "ontologies": ["obo:mondo","obo:efo"],
          "classes": ["MONDO:0000001","PATO:0000461"],
          "relations": ["rdfs:subClassOf"],
          "direct": false,
          "include_self": true
        }
      }
    },
    "required": ["disease"]
  },
  "object": {
    "alias": "MA456",
    "taxonId": 9606,
    "disease": "glioblastoma",
    "disease_id": "EFO:0000519"
  }
}
```

Response: [{"dataPath":"/disease_id","errors":["Provided term is not child of [http://purl.obolibrary.org/obo/MONDO_0000001,http://purl.obolibrary.org/obo/PATO_0000461]"]}]
